# Distributed Intelligent Learning and Decision Model Based on Logic Predictive Control

**DOI:** 10.1155/2022/6431776

**Published:** 2022-08-30

**Authors:** Yucheng Zhou, Wen Lu, Yingqiu Zhang

**Affiliations:** ^1^Chongqing Jiaotong University, Chongqing 400074, China; ^2^School of Sciences, Zhejiang SCI-TECH University, Hangzhou, Zhejiang 310000, China; ^3^School of Competitive Sports, Beijing Sport University, Beijing 100084, China

## Abstract

By the method of documentation and logical analysis, based on the data, based on logic and based on the knowledge of three kinds of artificial intelligence in the sports education, the intelligent learning system feedback delay are studied, combined with mobile communication which led to the artificial intelligence online sports games teaching, pattern recognition, and virtual technology combined with innovative teaching interaction and experience. Promoting the development of green PE teaching machine learning can identify the types of PE activities and realize efficient PE learning diagnosis. Intelligent decision support system can identify sports talents and improve the effect of personalized PE teaching evaluation. From the perspective of psychological development and education, the key problems to be solved in the integration of artificial intelligence and physical education are examined. Then, the consistent model predictive control for feedback delay of nonlinear sports learning multiagent system with network induced delay and random communication protocol is studied. Under the communication waiting mechanism designed, each agent has a certain tolerance of delay, and this tolerance can be determined by ensuring the stability of the system. At the same time, a random communication protocol is designed to ensure the ordered communication of the multiagent system. Finally, the effectiveness of the proposed algorithm is verified by numerical simulation. To solve the channel competition access problem of the sports intelligent learning system with special structure feedback delay model predictive control, a dual channel awareness scheduling strategy under the model predictive control framework was proposed, and the distributed threshold strategy of sensors and the priority threshold strategy of controllers were designed. It is proved that the sensor will eventually work at Nash equilibrium point under the policy updating mechanism, and the priority threshold strategy of the controller is better than the traditional independent and identically distributed access strategy. By avoiding the data transmission when the channel status is poor, the channel access of the system is efficient and saves energy.

## 1. Introduction

AI (artificial intelligence) technology is applied in training and competition stages, mainly through hardware terminals such as camera equipment and sensors for deep learning. In the process of sports training and competition, we maintain gentle state of mind which is a basic element, obtaining good results using machines to identify facial expressions in the video and image, and according to the facial features to predict detection machines, machine needs to face detection image processing tools and basic emotional states such as public data as the foundation; our country already has a mature machine for complex facial recognition task [1]. Foreign scholars use this method, using the tracking data, to construct the data-driven model. This model is mainly generated from the first person image reliable basketball action sequences, by way of unsupervised learning, and is widely used in the field of sports. Chinese scholars depend on the practical field of China sports development training, increase the intensity of research model, and understand the training and competition situation through artificial intelligence technology [2].

Networked model predictive control (NMPC) is a good method to deal with network induced delay and packet loss. The basic idea of model predictive control algorithm is to predict the future behavior of the controlled object based on the real-time state information of the controlled object, and the prediction is rolling forward; that is to say, the controlled object will re-predict once every period of time [3]. When the induced delay and packet loss occur in the controlled network, the system can iteratively optimize the received information according to the previous time, so as to find the optimal state sequence. For multiagent systems, when there is network induced delay or packet loss between agents, this method can also be used to compensate effectively. However, compensating the missing information directly with the feasible solution of model predictive control will inevitably bring errors, so using a waiting mechanism to deal with the missing information can ensure the authenticity of data.

At present, online sports game teaching is still inadequate, its teaching effect depends on the specific type of game, the game may induce distraction, and sustainability of the teaching effect needs to be verified. At present, “5G + physical education” is ready to take off. Artificial intelligence will rely on the characteristics of 5G technology, such as large connection, ultralow delay, and ultrahigh speed, to innovate the content and communication mode of physical education and further promote the innovation of sports game teaching scenes and teaching experience.v

## 2. Related Work

The combination of big data and mobile communication technology makes online physical education flourish. Mobile Internet game courses represented by serious games and sports games innovate physical education teaching methods and can promote students' physical and mental development in multiple dimensions. Currently, sports game courses integrate big data physiological measurements to optimize exercise intensity and exercise plan, which can be extended to family sports. Serious games refer to video games for the purpose of education, health, or scientific research, aiming to stimulate and develop the motivation of physical education and exercise habits. A large number of studies have proved that serious games have positive effects on academic knowledge, cognitive ability, professional and technical knowledge, learning motivation, and academic achievement [4, 5]. Foreign researchers developed the game “Virtual Physical Education Teacher,” which can design sports activities for different student groups based on big data feedback [6]. Sports games refer to games that combine body movement with electronic games based on motion sensor technology, represented by dance games. Because dance games can consume a lot of calories, they have been introduced into American youth sports courses and received positive feedback from parents and students. The skills acquired by young people in sports games can be transferred to promote physical, social, and cognitive development.

The combination of pattern recognition and model predictive control, feedback delay of sports intelligent learning system, augmented reality technology, and mixed reality technology provides a new window for the development of physical education technology. Pattern recognition technology captures and identifies movements through wearable devices to monitor physical education [7]. Sports courses with special requirements such as snow sports and golf break the limitation of teaching environment and reduce the risk of injury by combining the feedback delay of intelligent sports learning system with intelligent wearable devices and model predictive control. Augmented reality technology relies on wearable devices and obtains real-time multi-perspective image information through computer vision, so as to obtain 3D movement information of students and give evaluation. Mixed reality technology relies on wearable devices to achieve immersive physical education through user and environment interaction [8]. In addition, wearable devices can also be combined with virtual personal assistants to realize human-computer interaction in physical education teaching through emotional computing and cloud computing technology and develop personalized physical education teaching plans. The combination of pattern recognition and model predictive control with feedback delay of sports intelligent learning system can also meet the requirements of sports teaching for special groups. Recent studies have shown that the combination of brain science and model predictive control sports intelligent learning system feedback delay can stimulate the brain nerves of patients with lower body movement impairment, enabling them to imagine walking, obtain virtual movement experience, and promote rehabilitation [9]. At present, the combination of pattern recognition and model predictive control with feedback delay in sports intelligent learning system is still insufficient. For example, the teaching effect is limited by the type of sports skills. However, for open baseball, the migration effect of outfielders catching the ball is poor. At present, the feedback delay of model predictive control sports intelligent learning system may be more suitable for closed sports skill learning, and its ecological validity needs to be improved.

Machine learning is mainly used for intelligent classification and prediction. At present, machine learning is mostly used in competitive sports, and its influence on physical education is mainly reflected in two aspects. First, deep learning algorithms can be used for sports activity type recognition. For example, artificial neural networks can assess individual exercise metabolic equivalents and determine individual activity types (low intensity activity, exercise, vigorous exercise, and housework/other activities). Computational modeling can monitor and feed back muscle state in real time to predict fatigue and avoid sports injuries. Second, deep learning algorithm can be used for PE learning diagnosis and performance prediction. By mining the historical data of training and competition to predict the results of the competition [10], it provides data basis for hierarchical physical education. At present, PE learning diagnosis requires high precision and accuracy of deep learning algorithm, and its accuracy needs to be optimized.

Model predictive control algorithm is an advanced control technology developed recently [11]. The initial form of model predictive control is dynamic matrix control [12], model predictive heuristic control [13], etc. In [14], the generalized model predictive control was proposed by researchers. However, due to the underdeveloped science and technology at that time, model predictive control (MPC) is difficult to be widely used in industry due to its large amount of computation. Until the information age, the relevant theories of model predictive control have been improved, so the examples closely combining model predictive control algorithm with practical application also show diversity [15]. Model predictive control with other optimization control algorithm: model predictive control for the cost function is to optimize the real time updated in each moment will be predicted, which is often said that it will roll forward in time domain of a time step, so the model predictive control is also known as rolling time domain control. Compared with traditional control methods, the special characteristics of model predictive control can be summarized as four points [16]. Finally, model predictive control has the ability to explicitly deal with all kinds of constraints of the system. In model predictive control, constraint conditions are written into the optimization problem in the form of mathematical expressions and solved directly in a mathematical way, so as to obtain the control quantity and state quantity satisfying the constraints. Therefore, model predictive control has attracted much attention in recent years [17, 18].

So far, comprehensive research on model predictive control at home and abroad was carried out. Model predictive control is roughly divided into three types: centralized model predictive control, decentralized model predictive control, and distributed model predictive control. In [19], a centralized model predictive controller is designed for multiagent systems. However, the decentralized model predictive control mentioned in [20] is based on a decentralized model, which only contains the input and output information of each agent without information interaction between agents [21]. Distributed model predictive control allows communication and information sharing among agents to achieve global optimality of agent systems. Therefore, DMPC reduces the complexity of optimization problems and improves work efficiency. In addition, model predictive control can not only deal with multiple constraints, but also calculate the control input sequence through online solution [22, 23]. The prediction and evaluation results are mainly nonlinear models based on machine learning algorithms. This is to improve the fitting ability of the regression model and accurately predict the explained variables. A prediction model of sports performance based on machine learning algorithm is proposed [24]. Its working principle is to improve the status quo of performance prediction, improve the evaluation level of physical fitness, and make the prediction results accurate by using the principle of structural risk minimization.

## 3. Feedback Delay Diagnosis of Sports Intelligent Learning System Based on Model Predictive Control

### 3.1. Sports Intelligent Learning Feedback Delay Diagnosis

**Figure 1:**
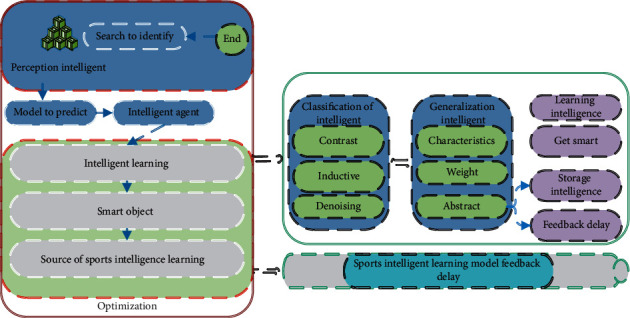
Time-delay frame diagram of sports intelligent learning feedback.

Machine learning is mainly used for intelligent classification and prediction. At present, machine learning is mostly used in competitive sports, and its influence on physical education is mainly reflected in two aspects. First, deep learning algorithms can be used for sports activity type recognition. For example, artificial neural networks can assess individual exercise metabolic equivalents and determine individual activity types (low intensity activity, exercise, vigorous exercise, and housework/other activities). Computational modeling can monitor and feed back muscle state in real time to predict fatigue and avoid sports injuries. Second, deep learning algorithm can be used for PE (physical education) learning diagnosis and performance prediction. By mining the historical data of training and competition, we can predict the result of the competition and provide data basis for stratified physical education. At present, PE learning diagnosis requires high precision and accuracy of deep learning algorithm, and its accuracy needs to be optimized. The time-delay frame diagram of sports intelligent learning feedback is shown in Figure 1.

As shown in Figure 1, model predictive control is an important development direction in sports training activities, which mainly refers to the simultaneous acquisition, processing, editing, storage, and display of two different types of information media technologies. Multimedia computer-assisted instruction needs to be based on the artificial intelligence technology and complex program; in the process of sports training, multimedia computer technology is used to realize the three-dimensional model, and the plane model interacts and promotes the teaching effect. The main purpose is to provide different stimulus in the process of sports training for athletes, control direct teaching environment to keep their nervous system excitability, make them interested in sports, help athletes broaden their horizons, train athletes' intelligence, and promote the development of athletes' personality, with greater superiority. Multimedia computer aided instruction belongs to the new trend of sports technology development, which effectively creates a relaxed environment for athletes and makes the feedback delay of sports intelligent learning system play a practical role.

Artificial intelligence technology has been applied in athlete training. Statistics of sports training are based on numbers, and automatic digital report is the development direction of sports training, which can be realized only by artificial intelligence technology. Artificial intelligence technology can completely change live broadcasting. According to sports events, artificial intelligence technology can choose the right successive angle, understand the real-time situation of athletes, and timely find the emergency situation in the training process.

It is an effective way to solve dynamics problems by using neural network intelligent technology. Dynamics is an important content in sports training. Dynamics mainly uses dynamic data during the execution and separation of 90-degree cutting movements to provide theoretical support for athletes' sports training plans. The principle of neural network intelligent technology is to use feedforward neural network to predict the torque and ground response of joints, knee joints and manic joints, and record the trajectory and joint angle as the input data of neural network intelligent technology. In the actual process of sports training, the training data and prediction data of athletes have a strong correlation in the process of sports. The joint angle of an athlete is used as an input parameter. The higher the prediction accuracy of joint torque, the higher the prediction accuracy of ground reaction force. Through the use of inertial sensors, the rapid changes of movement direction and mechanical parameters can be predicted to help the athlete improve the sports training program.

### 3.2. Research on Model Predictive Control Algorithm

**Figure 2:**
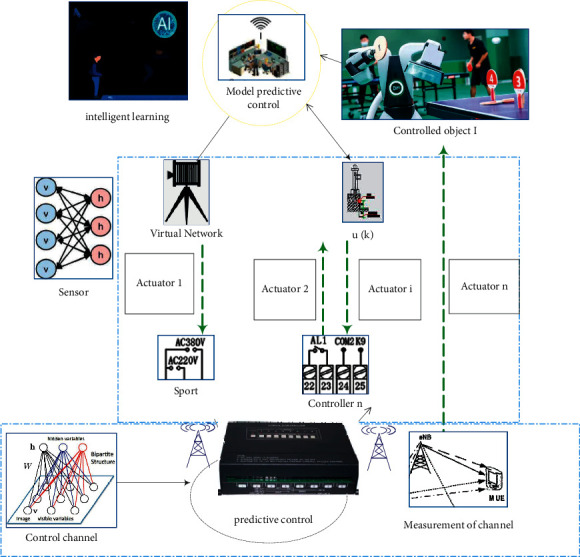
Structure of sports intelligent learning system based on model predictive control.

The structure of the sports intelligent learning system of model predictive control is shown in Figure 2, in which *n* independent control systems are controlled by remote controllers installed on the cloud computing platform, and the sensing data and control data are transmitted through wireless channels. The channel from the sensor side to the controller side is called the measurement channel, and the channel from the controller side to the actuator side is called the control channel.

There are two main reasons for packet loss in the sports intelligent learning system based on model predictive control. One is packet collision; that is, the wireless channel allows only one user access at most at any time, and the simultaneous access of multiple users will lead to transmission failure of all users. The other is transmission error, where packets may be discarded due to errors in transmission due to interference and signal fading.

The cost function is as follows:

(1) $$\begin{array}{c} R\left(x\left(n\right),y\left(n\right)\right)=\left.\left|x\left(n\right.\right|k\right)-y{\left.\left(n|k\right)\right|}^{2}\text{.} \end{array}$$

In the standard of rolling optimization mechanism, inequality is usually meet; however, if the given reference trajectory is a cycle of time-varying reference trajectory, it may be a sudden change, so it is difficult to find a suitable weighting matrix to ensure inequality always meet, and inequality cannot be sure; it is difficult to prove the system stability. Therefore, a time-varying weight matrix is considered in this section to ensure the inequality. Design the following corresponding terminal cost function, terminal domain, and local controller:

(2) $$\begin{array}{c} V_{j}\left(n\left|k|\text{)}|=\right|\right.x\left(n\right)-v{\left.\left(n|k\right)\right|}^{2}\text{.} \end{array}$$

The model predictive control algorithm is shown in Algorithm1:.

In sports training activities, the musculoskeletal system injury of athletes is more serious, and artificial intelligence technology can be used to monitor the quality of human movements. In the process of movement analysis of tennis special sports technology, AI technology is the future development direction of tennis training. Tennis needs to be separated according to the technology and movement of the sport purpose, and the important content of each subitem is determined. The key direction is to establish excellent technical model, based on the technical movement parameter data of excellent athletes. The weighted value of node connection in the second part represents the connection signal. The output mainly depends on the network connection mode, and the weighted value changes with the output function. In motion learning, the artificial neural network is used to record data for many times to obtain the motion sequence, and the neuron output is used. It is helpful to analyze the movement pattern of athletes.

The combination of expert system and decision support system can form intelligent decision support system, which can assist in solving the decision-making and evaluation problems of physical education. Intelligent decision support system can be used for personalized sports training. The expert system of hurdle sports can generate the training load of hurdle sports and predict the result. There are also studies on the use of big data mining and analysis, analysis of learning preferences, and learning strategies, to achieve personalized physical education. Intelligent decision support system can be used for sports material selection.

## 4. Sports Intelligent Learning System Feedback Delay

From the basic framework of sports intelligent learning system, we can know that predicting the behavior of other agents plays a very important role in the learning results of agents. Classical *Q* learning assumes that the environment is static, so model-free reinforcement learning can be adopted. However, for agent *I*, the generalized environment is dynamic, so model-free learning cannot be adopted. Model-based reinforcement learning learns the optimal strategy by modeling the environment. Because it can reflect the characteristics of the environment in time and the state prediction mechanism can reflect the dynamics of other agents, the combination of the two can build a model that reflects the dynamic environment, namely, the generalized environment. Based on the above ideas, if the environmental dynamics can be accurately and timely estimated and predicted, then, to a certain extent, the model of generalized environment is accurate and stable. Therefore, the generalized environment can be regarded as a nearly static environment, and then the optimal strategy can be obtained by dynamic programming method.

Dynamic corresponding agent: because the environment is caused by other intelligent beings, as a result, accurate and timely strategy to estimate the other agent and the dynamic characteristics of the environment to be effective are modeled, and then, using the reinforcement learning for optimal decision to adapt to the dynamic characteristic, the comparison is carried out with the classical control theory of tracking control. Before designing the state prediction mechanism, the basic definition of state prediction is given.

(3) $$\begin{array}{c} P\left(x,a\right)=\left(1-\lambda \right)P\left(x,a^{j}\right)\text{.} \end{array}$$

In the feedback delay framework of sports intelligent learning system based on model predictive control, the *Q* function will become a dynamic process due to the existence of other agents. This means that due to the change of other agents' strategies, the optimal action in the current state may become the worst action in the next experience, and the corresponding *Q* table will also change. However, this situation does not exist in single-agent reinforcement learning and multiagent learning with joint states and joint actions. Therefore, the action selection mechanism mentioned above cannot be used to deal with the dynamic environment in which the feedback delay framework of the model predictive control sports intelligent learning system exists. Otherwise, action selection oscillations will occur in the agent's behavior, and even the agent cannot complete the task in an effective time. Therefore, a new action selection mechanism needs to be designed. The improved action selection mechanism fully considers the dynamic nature of the environment and can effectively guide agents to balance the “exploration-utilization” problem. See Algorithm 2.

AI can change the structure of the brain and improve cognitive performance. For example, action video games using computer vision can enhance attention, improve multi-objective tracking, and speed up reaction times. AI can also boost motor cognitive processing and improve performance. For example, electroencephalography can be used to explore brain activity during exercise. Neurofeedback training can improve attention and working memory, so as to develop soccer shooting skills and archery skills.

There is a spiral relationship between the application of artificial intelligence and cognitive development, and the two promote each other. Smart PE teaching should conform to students' cognitive level, consider the potential impact of pulse technology on cognitive development, timely adjust teaching requirements, and achieve accurate teaching. Follow-up research should focus on the role of artificial intelligence in the development of sports core literacy and focus on exploring the relationship between artificial intelligence, cognition, and physical education, so as to provide a cognitive perspective for in-depth analysis of the mechanism of artificial intelligence in physical education.

Artificial intelligence promotes the development of ubiquitous learning and adaptive learning, and physical education is further personalized and fragmented, which brings the difficulty of monitoring the teaching effect. The contradiction between personalized learning and collectivized teaching system is becoming more and more prominent. Objectively, it is necessary to constantly upgrade technical monitoring means and provide accurate and systematic big data monitoring support services; subjectively, physical education teachers should combine the students' personality characteristics, reconstruct the teaching content and the teaching process, and seek a multi-teaching evaluation method combining individuation and collectivized learning.

In sports, key note recognition is an important content of sports analysis, which provides effective sports analysis and auxiliary analysis for athletes. Initially, it mainly tracks each individual key gesture, using the characteristics of every athlete research group. In the later development process, its main purpose is to group the image processing and sampling and access global information and processing; data were collected to exceptions and are classified. The traditional technology has been unable to meet this requirement.

## 5. Example Verification

Because the feedback delay algorithm of model predictive control sports intelligent learning system adopts model-based reinforcement learning, Friend-Q belongs to model-free reinforcement learning. Generally speaking, model-based reinforcement learning has higher learning efficiency than model-free reinforcement learning because it records the learning experience of agents and can make full use of such historical information. In order to ensure the consistency of the comparison between the two algorithms, Friend-Q is implemented by model-based reinforcement learning, so that both algorithms belong to model-based reinforcement learning algorithm.

Similarly, in order to ensure the consistency of the premise of comparison as much as possible, the parameter settings with the same meaning in the above two algorithms are also the same, including the discount coefficient of 0.8, the threshold of 0.7, and the prediction function RME of 0.8. The experimental results are repeated 50 times, and the average value is taken.

**Figure 3:**
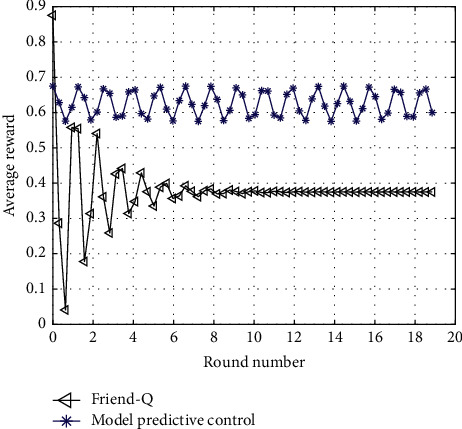
Average predictive control reward obtained by completing the task.

**Figure 4:**
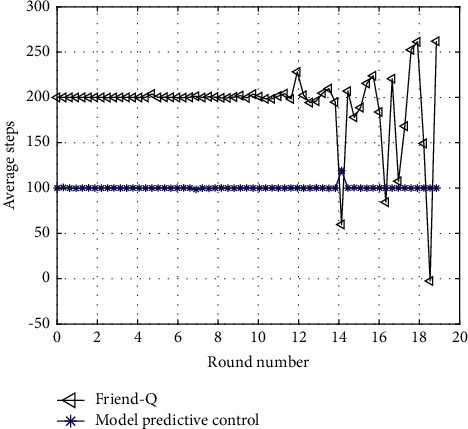
Average predictive control steps obtained by completing the task.

The other two areas of comparison are the average number of steps required to reach the target state and the average reward earned. As shown in Figures 3 and 4, the feedback delay algorithm of the sports intelligent learning system based on model predictive control can learn the optimal tracking strategy within shorter learning steps and obtain higher rewards at the same time. Therefore, the learning speed of the feedback delay of the sports intelligent learning system based on model predictive control is obviously faster than that of Friend-Q. In addition, the feedback delay of the sports intelligent learning system of model predictive control can learn the optimal strategy and reach the target state with a minimum of 7 steps (excluding the last step when reaching the target).

**Figure 5:**
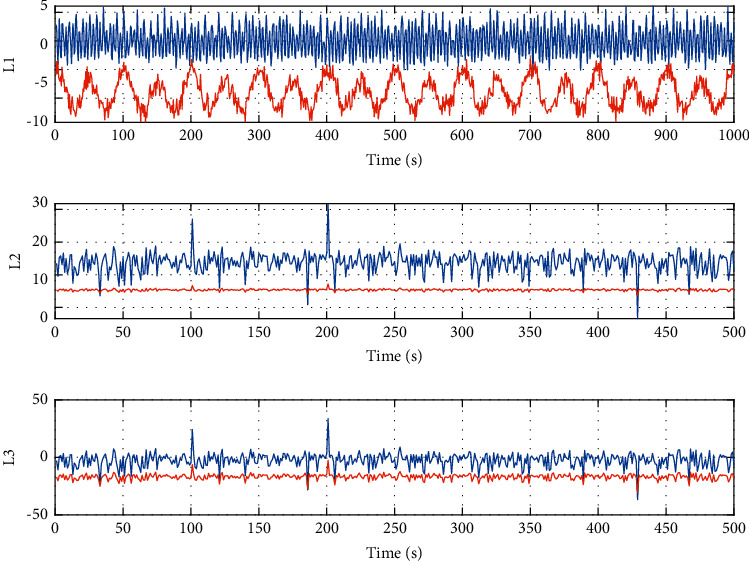
State response curve (the blue line is generated by the algorithm in this section; the red line does not have any control compensation scheme).

**Figure 6:**
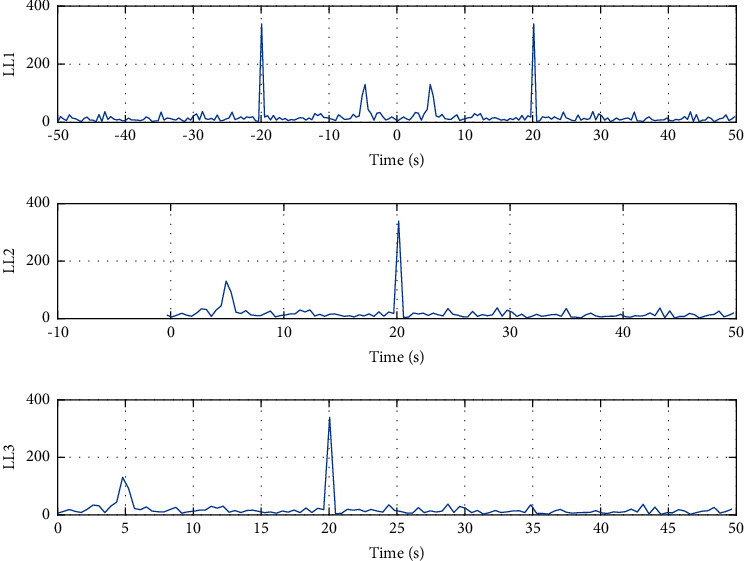
The real control object input of the system under the model pre-side control algorithm.

Figures 5 and 6 show the state response curve and real control input curve under the distributed access mechanism of sensors, the random priority threshold strategy of the controller, and PBMPC algorithm of the model in the measurement control and sports intelligent learning system. All systems are found to be stable and satisfy control constraints.

**Figure 7:**
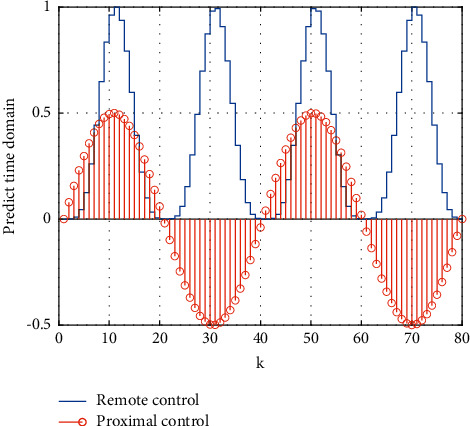
Time-domain variation curve of feedback delay of sports intelligent learning system based on model predictive control.

**Figure 8:**
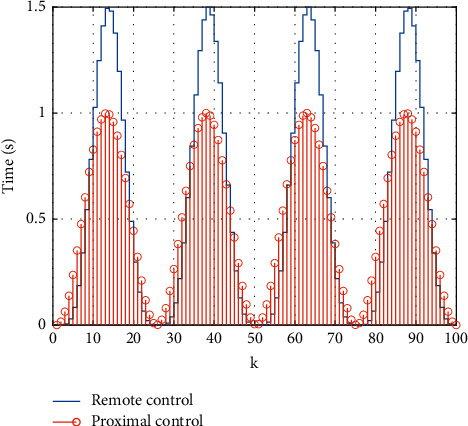
Solution time of feedback delay of sports intelligent learning system based on model predictive control.

Compared with the networked fixed time domain, the efficiency of the control scheme can be verified. The simulation results can be seen in Figure 7 which shows that the time-varying prediction time domain obtained by the feedback delay algorithm of the model predictive control motion intelligent learning system is good.  Figure 8 shows the calculation time of each step of the two control schemes and evaluates the computational complexity of the algorithm accordingly.

The feedback delay of sports intelligent learning system based on model predictive control belongs to the computer system simulation technology for creating and experiencing virtual world. It mainly integrates computer graphics, multimedia technology, artificial intelligence technology, and man-machine interface technology to provide support for creating and experiencing virtual world. The feedback delay of the model predictive control sports intelligent learning system enables athletes to have a deep understanding of sports with the help of virtual technology. In the virtual environment, athletes can get a change from perceptual rationality, so that they can have a deep understanding of sports spirit, actively explore and collect information, and develop their imagination ability. Sports intelligent learning system feedback delay of model predictive control in the process of sports training has a wide range of applications, for example, establishing physical exercise prescription sites and analyzing the practical development of sports. A three-dimensional intelligent learning system with feedback delay is established by using model predictive control of motion, and a human motion model is established to make robots and athletes find problems in the process of motion. Thirdly, the feedback delay of the model predictive control sports intelligent learning system is simulated to the real world, and it is used to surpass the real world, so that the athletes can achieve the goal of high efficiency in the virtual environment, enhance the athletes' experience of the virtual environment, and strengthen their understanding and mastery of sports.

## 6. Conclusion

In the process of integrating artificial intelligence technology into sports, the sports effect has made continuous progress, and artificial intelligence technology has been developing continuously. At present, important breakthroughs have been made in basketball, tennis, and skiing, and it is an important development direction to integrate artificial intelligence technology into sports. Data packet loss between agents is described by distribution, and compensation is made by using predictive sequence data loss of model predictive control. In order to avoid chaotic communication between agents, the largest prediction time domain is chosen as the next prediction time domain of the whole multi-intelligence system at each update moment. Finally, a numerical example is given to verify the effectiveness of the proposed algorithm. The integration of physical education and artificial intelligence can promote the construction and interaction of massive physical education resources, innovate teaching methods and interaction, provide physical education learning diagnosis and talent identification, promote the reform of physical education, and cultivate innovative sports talents. The future development of intelligent physical education requires that we should attach importance to the spiraling interaction between artificial intelligence and students' cognitive development, comprehensively consider the advantages of personalized and collectivized learning, dynamically examine teachers' information literacy, promote teachers' empowerment, and support multidimensional evaluation of students' physical ability. Condition forecasting method used in this study is based on the accurate observation of other agent history behavior after the forecast; in some practical applications, to the presence of large amounts of agents in the environment or obstacles leads to an agent's behavior observation which is not accurate, so we cannot update forecast function because of the deviation of decision-making. Therefore, the next step is to design a more robust state prediction method in order to improve the robustness of the whole system decision.

## Figures and Tables

**Algorithm 1:**
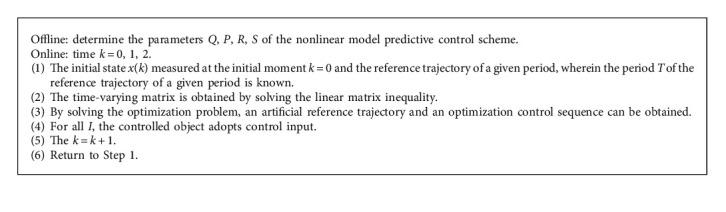
Overview of nonlinear model predictive control algorithm.

**Algorithm 2:**
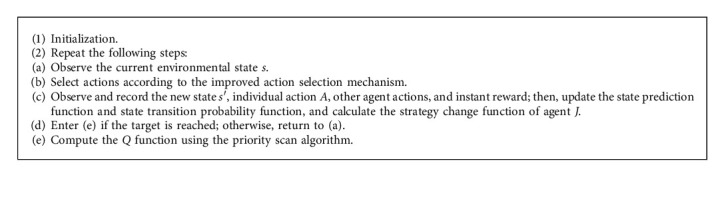
Model prediction action selection mechanism algorithm.

## Data Availability

The data used to support the findings of this study are included within the article.
